# Laundering CNV data for candidate process prioritization in brain disorders

**DOI:** 10.1186/s13039-019-0468-7

**Published:** 2019-12-26

**Authors:** Maria A. Zelenova, Yuri B. Yurov, Svetlana G. Vorsanova, Ivan Y. Iourov

**Affiliations:** 1 Mental Health Research Center, Russia Moscow, 115522; 2 Academician Yu.E. Veltishchev Research Clinical Institute of Pediatrics, N.I, Pirogov Russian National Research Medical University, Ministry of Health of the Russian Federation, Russia Moscow, 125635

**Keywords:** Autism, Bioinformatics, Brain, CNV, Intellectual disability, Pathways

## Abstract

**Background:**

Prioritization of genomic data has become a useful tool for uncovering the phenotypic effect of genetic variations (e.g. copy number variations or CNV) and disease mechanisms. Due to the complexity, brain disorders represent a major focus of genomic research aimed at revealing pathologic significance of genomic changes leading to brain dysfunction. Here, we propose a “CNV data laundering” algorithm based on filtering and prioritizing of genomic pathways retrieved from available databases for uncovering altered molecular pathways in brain disorders. The algorithm comprises seven consecutive steps of processing individual CNV data sets. First, the data are compared to in-house and web databases to discriminate recurrent non-pathogenic variants. Second, the CNV pool is confined to the genes predominantly expressed in the brain. Third, intergenic interactions are used for filtering causative CNV. Fourth, a network of interconnected elements specific for an individual genome variation set is created. Fifth, ontologic data (pathways/functions) are attributed to clusters of network elements. Sixth, the pathways are prioritized according to the significance of elements affected by CNV. Seventh, prioritized pathways are clustered according to the ontologies.

**Results:**

The algorithm was applied to 191 CNV data sets obtained from children with brain disorders (intellectual disability and autism spectrum disorders) by SNP array molecular karyotyping. “CNV data laundering” has identified 13 pathway clusters (39 processes/475 genes) implicated in the phenotypic manifestations.

**Conclusions:**

Elucidating altered molecular pathways in brain disorders, the algorithm may be used for uncovering disease mechanisms and genotype-phenotype correlations. These opportunities are strongly required for developing therapeutic strategies in devastating neuropsychiatric diseases.

## Background

Brain disorders frequently result from genomic variations altering a variety of molecular and cellular pathways [1]. Due to a significant overlap between genetic variations associated with phenotypic spectrum of various disorders, psychiatric genetic research may be focused on interactomes (networks of interacting genes/proteins) influencing certain pathways. This is further supported by the findings indicating an increase of total burden of rare, inherited or de novo copy number variations (CNVs) to be associated with psychiatric disorders [2], suggesting that different malfunctioning genes might be involved in the same biological process, disruption of which causes the disease. Protein-protein interaction (PPI) networks (molecular pathways) seem to be a more reliable drug target than gene mutations or CNV per se. Indeed, molecular pathways to intellectual disability (ID), autism spectrum disorders (ASD) and schizophrenia are repeatedly reported to be based on specific PPIs [1, 3]. The convergent pathways include, but are not limited to, those regulating neurogenesis, neuronal migration, synaptic functions, transcription, translation, cell cycle and programmed cell death [1, 4]. The majority of the networks altered in brain diseases regulate either processes crucial for neural development and functioning or those influencing cell cycle and communication. Particularly, RhoGTPase pathway is involved in nervous system development, dendritic spines formation and neuronal differentiation [5]; Ras/RAP pathway is responsible for long term potentiation of AMPA receptors (Ras) and long term depression (Rap) [6]. Cell cycle pathway may be altered to produce genome instability leading to cancer or neurodegenerative diseases [7]. More precisely, ERK/PI3K signaling pathway influences the more general pathway regulating the cell cycle and cell differentiation and is altered in neurodevelopmental diseases [8]. Wnt signaling pathway takes part in neuronal migration, dendrite and synapse formation, as well as axon guidance [9]. However, the alterations to these pathways are rarely addressed in the CNV context, probably due to the lack of appropriate bioinformatic algorithms [10, 11]. Here, we propose an algorithm for “laundering” CNV data based on a previously described bioinformatic technique for CNV prioritization [12]. The algorithm may be applicable for identifying causative (candidate) processes for brain disorders in diagnostic and basic research.

## Methods

**Fig. 1:**
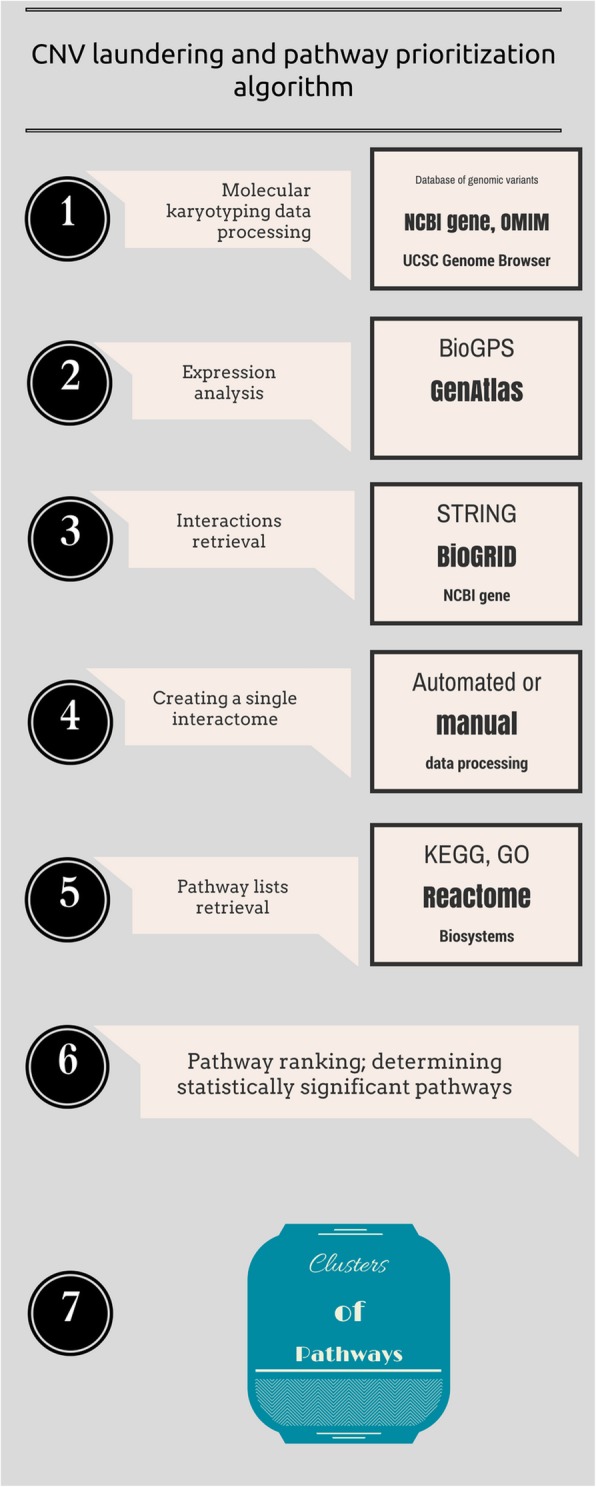
Data laundering algorithm for CNV prioritization

We propose a CNV prioritization algorithm — “data laundering” — suitable both for diagnostic and basic research. The algorithm is based on an idea that brain diseases result from genomic alterations affecting directly the brain [13, 14] and, consequently, predominant expression of a gene in the central nervous system increases the probability of its contribution to a neurobehavioral phenotype [12]. We designate the algorithm as “laundering” because of the resemblance to machine-washing (each step processes the data from the previous stage to be filtered several times using different criteria). Figure 1 schematically outlines the procedure.

First, a pool of CNVs is obtained by molecular karyotyping. At this stage, CNVs are checked for recurrence by in-house and web databases. In-house databases of genomic variants obtained by similar microarray types are applied to spot recurrent aberrations. It is to note that the indexation of CNV in a Database of Genomic Variants or any other database dedicated to non-pathogenic genome variations is not a criterion for the exclusion at following stages. Further, the localization and ontology of CNV genes (e.g. using UCSC genome browser, NCBI gene, OMIM, PubMed etc.) are obtained. At this stage, genes lacking appropriate ontology, CNV encompassing introns, recurrent/non-pathogenic CNV are excluded from further analysis. It is worth mentioning that here, CNV are defined as copy number DNA gains/losses < 500 kbp.

Secondly, the genes are in silico analyzed in terms of the expression in the central nervous system. As brain pathology is suggested to be mainly associated with neurobehavioral phenotypes, it is recommended to proceed to the next step with a pool of genes highly expressed in the brain.

Third step is referred to as retrieving gene-gene interactions. Considering the differences in databases, it is suggested to use several resources (e.g. NCBI gene, BioGRID, STRING). Here, we have merged data from NCBI gene, BioGRID and STRING.

During the fourth step, the gene list is evaluated for uncovering interactions and interaction enriched gene clusters (sets of interacting genes). Further, only large groups of interacting genes are analyzed, leaving aside small clusters of interacting elements. This criterion is based on a hypothesis that highly interacting genes (proteins) are more likely to be involved in the same processes or influence a disease with similar symptoms [15].

Fifth, the pathway lists are obtained for the set of interacting genes. During database selection, one should consider such parameters as the nature and curability of pathway data. Here, Gene Ontology (GO), KEGG, Reactome, NCBI Biosystems were used.

Sixth, to process the pathway lists, we introduce a parameter (prioritization criterion) to determine significantly enriched pathways. To calculate the parameter, a total number of genes for each pathway are obtained. Pathways, in which less than 25 genes are affected by CNVs, are excluded. The remaining pathways are ranked using the index of pathway prioritization (I_PP_):

$$ {I}_{PP}=\frac{\sum {N}_{CNV\  genes}}{\sum {N}_{pathway\ genes}} $$

where *I*_*PP*_ — index of pathway prioritization; *N*_*CNV genes*_ *—* number of CNV genes in a pathway found in molecularly karyotyped cohort; *N*_*pathway genes*_ — total number of pathway genes. If the I_PP_ is higher than average (i.e. evaluated by three sigma rule), the pathway is prioritized.

Seventh, ontologies attributed to the elements of prioritized pathways are considered; pathways are clustered according to the involvement in shared networks (cascades of processes) [16]. Thus, the algorithm provides a set of enriched processes (clusters of pathways) in a disease or in an individual patient.

**Fig. 2:**
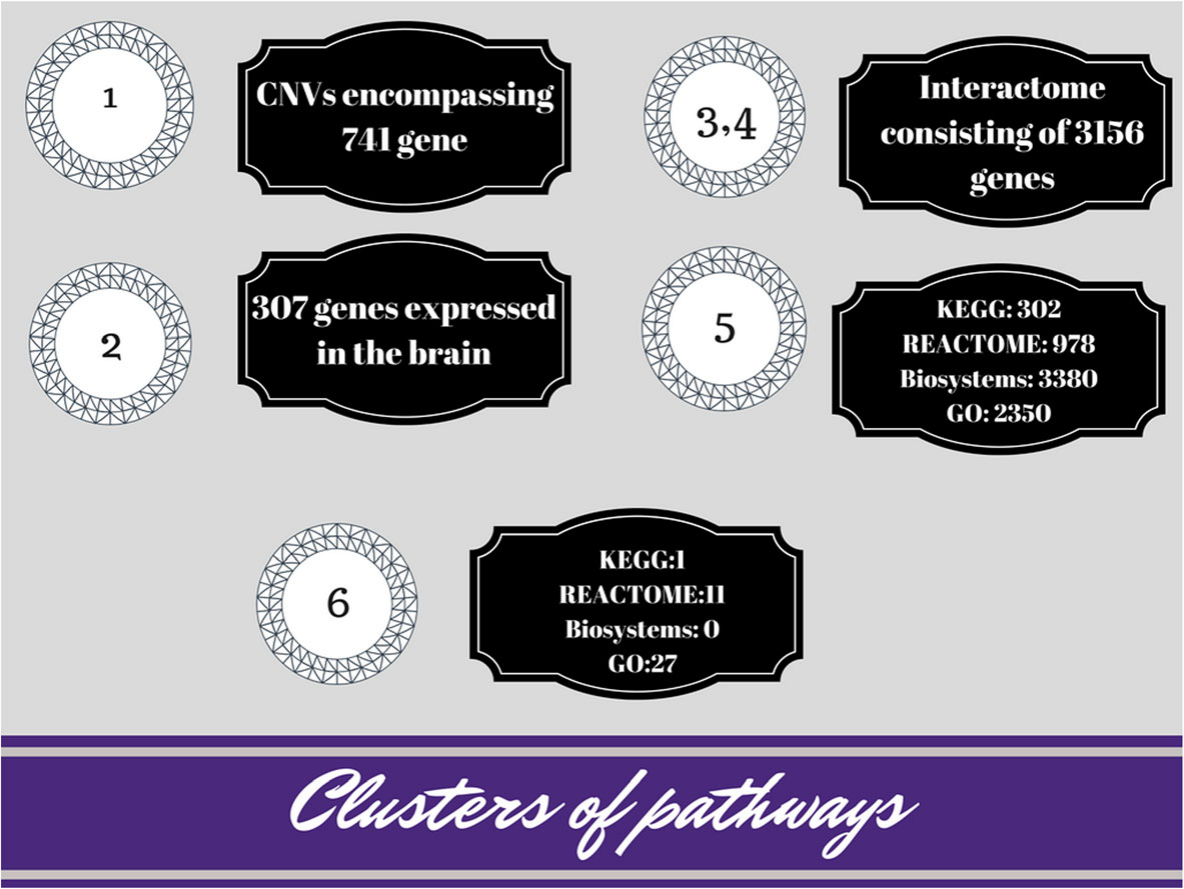
Intermediate results before pathway clustering

Using the algorithm and Affymetrix CytoScan HD microarray, we analyzed 191 genomes (DNA isolated from peripheral blood) of children with ID, ASD and congenital abnormalities without gross chromosomal and genomic rearrangements (i.e only the CNVs less than 500 kbp in size were included). The raw results of the algorithm processing are shown in Fig. 2.

## Results

We obtained a set of 741 genes affected by pathogenic or likely pathogenic CNVs. “Expression filtering” allowed to select 307 genes highly expressed in the brain. Cross-checking interactions of these genes (step 3) was used for building an interactome (step 4) encompassing 3156 genes. These genes were involved in 302, 978, 3380, and 2350 pathways, according to KEGG, REACTOME, Biosystems, and GO, respectively. For each pathway, we calculated I_PP_, which allowed us to obtain enriched pathways for each database: KEGG — 1, REACTOME — 11, Biosystems — 0, GO — 27. Pathway clustering was performed according to pathway ontologies.

The application of CNV prioritization or “data laundering” algorithm yielded 39 genomic networks (pathways) forming 13 clusters of processes, involving 475 genes. These pathway clusters were as follows: neurodegenerative diseases, proteasome, signaling by ERBB4, transcription regulation, regulation of TP53, signaling by NOTCH, senescence, mitosis, DNA repair, vesicles functioning, actin functioning, macromolecular interactions, B cells functioning (Table 1).

**Table 1:** Pathways organized by clusters

Cluster name	Pathways
Neurodegenerative diseases	● Neurodegenerative diseases
Proteasome	● Downregulation of TGF-beta receptor signaling
Signaling by ERBB4	● Signaling by ERBB4
	● Nuclear signaling by ERBB4
Transcription regulation	● SMAD2/SMAD3:SMAD4 heterotrimer regulates transcription
	● RNA polymerase II repressing transcription factor binding
	● RNA polymerase II activating transcription factor binding
Regulation of TP53	● Regulation of TP53 degradation
	● Regulation of TP53 activity through acetylation
	● p53 binding
Signaling by NOTCH	● Activated NOTCH1 transmits signal to the nucleus
	● Signaling by NOTCH2
Senescence	● Oncogene induced senescence
Mitosis	● Mitotic cytokinesis
	● Spindle pole
DNA repair	● HDR through single strand annealing (SSA)
	● Nucleotide-excision repair, DNA damage recognition
Vesicles functioning	● SNAP receptor activity
Actin functioning	● Stress fiber
	● Podosome
Macromolecular interactions	● Insulin receptor binding
	● Protein kinase C binding
	● Fibroblast growth factor receptor binding
	● Core promoter sequence-specific DNA binding
	● RNA polymerase II core binding
	● Beta-amyloid binding
	● Epidermal growth factor receptor binding
B cells functioning	● B cell homeostasis
	● B cell apoptotic process

## Discussion

According to the value of *Ipp*, the most significant pathways clusters were “proteasome”, “neurodegenerative diseases”, “regulation of TP53”, “vesicles functioning”, “signaling by NOTCH”, “actin functioning”. Proteasome cluster was the most enriched one. Alterations to the proteasome complexes decrease proteolytic activity leading to the accumulation of damaged or structurally abnormal proteins. Similar protein accumulation may underlie neurodegenerative, cardiovascular and autoimmune diseases [17]. Neurodegenerative diseases cluster was enriched in genes associated with several devastative diseases and implicated in a variety of molecular/cellular processes. More precisely, *CDK5* is involved in synaptic plasticity and neuronal migration; *DCTN1* takes part in the formation of mitotic spindle and axons; *FUS* regulates gene expression and maintains the integrity of the genome; *GRN* regulates cell growth, and *OPTN* participates in membrane transport [18]. The p53-pathway consists of genes that respond to a wide range of stress signals. Stress responses include apoptosis, cellular senescence and cell cycle arrest. In addition, p53-regulated genes may produce proteins that transmit stress signals to neighboring cells and. These genes are involved in DNA reparation, regulation of p53 and binding to signaling pathways [19]. Disruption of synaptic vesicles is associated with developmental disorders. The fusion of synaptic vesicles with a presynaptic plasma membrane, followed by the release of a neurotransmitter, is essential for the neural transmission [20]. The proteins belonging to the SNARE complex (Synaptic-soluble N-ethylmaleimide-sensitive factor attachment receptor) participate in the majority of membrane-vesicles fusion events. A number of diseases are associated with mutations in the genes of this complex; for example, homozygous mutations of *SNAP29* leading to impaired endocytic recycling and cell motility has been associated with CEDNIK syndrome (cerebral dysgenesis, nervous system disorders, ichthyosis and palmar-plantar keratodermia) [21]. Additionally, a decrease of SNAP25 was found in the hippocampus of patients with schizophrenia. A single nucleotide polymorphism (SNP) in *SNAP25* was associated with hyperactivity in ASD. In high-functioning autism, increased syntaxin 1A expression was observed. Various studies showed that the reduced expression in the anterior part of the cingulate gyrus was observed in patients with ASD [22]. Notch signaling plays a significant role in embryonic development and dendritic development. In mammals, deletions of the Notch signal modulator (Numb) disrupted the maturation of neurons in the developing cerebellum, and violated axon branching in sensory ganglia [23]. The dysfunction of the signaling pathways that reorganize synaptic actin is associated with a variety of brain development abnormalities, including ASD, schizophrenia and ID. Indeed, genes such as *SHANK3, GIT1, DISC1, SRGAP3, OPHN1, LIMK1, NRG1, CYFIP1, SYNGAP1, KALRN, NCKAP1* and *CNKSR2* regulate upward signaling that stimulate the dynamics of the actin cytoskeleton in dendritic spines [24].

Currently, individual genome analysis obtains big data which are to be processed for basic, diagnostic and therapeutic purposes. Molecular karyotyping detects CNVs, which may output candidate gene and pathway lists. To discover genetic basis of an individual’s phenotype or pathways to a disorder, it appears necessary to answer two questions:

1. What are the pathways disrupted by CNV genes?
2. Do these pathways merge into a single global cluster reflecting a specific cellular/molecular process?

Application of bioinformatic strategies similar to the data laundering algorithm is able to answer these questions. It is necessary to stress that data analysis requires tools considering multiple factors and a theoretic background. Pathway clustering represents a promising bioinformatic tool, enabling the development of therapeutic strategies based on a molecular mechanism [25]. Similarly, our algorithm may be used as for an individual, particularly, as for a disease, as a whole. Furthermore, “data laundering” method is based on freely available web tools.

## Conclusion

The algorithm has been applied to a cohort of 191 children with ID, ASD and congenital abnormalities, yielding 13 pathway clusters potentially associated with brain disorders: neurodegenerative diseases, proteasome, signaling by ERBB4, transcription regulation, regulation of TP53, signaling by NOTCH, senescence, mitosis, DNA repair, vesicles functioning, actin functioning, macromolecular interactions, B cells functioning. Thus, the data laundering algorithm using CNV data allows obtaining clusters of candidate (disease-associated) processes. This algorithm is important for further basic and diagnostic research. Moreover, the application of the algorithm to molecular diagnostics of genomic pathology makes it possible to expand our knowledge about disease mechanisms in individual cases. Our findings may have importance for the development of therapeutic strategies and relevant psychological intervention for genetically determined ID and ASD cases caused by CNVs [26–28]. Molecular pathways are key elements of etiological concepts in brain disorders, significantly contributing to our understanding of neurological and psychiatric diseases. To determine disease mechanisms, one has to uncover the molecular and cellular pathways in addition determining a gene or chromosome abnormality underlying the condition. In other words, the main task for such studies is to find disrupted biological processes, which should be properly reflected in common disease description [29–32]. The application of our algorithm can lead to successful identification of molecular and cellular mechanisms for brain diseases for developing personalized therapeutic strategies.

## Data Availability

The datasets used and analyzed during the current study are available at http://dekanat.bsu.edu.ru/f.php/1/disser/case/filedisser/filedisser/998_dissertaciya_zelenova.pdf

## Abbreviations

ADHD: Attention deficit hyperactivity disorder
ASD: Autism spectrum disorder
CNV: Copy number variation
DNA: Deoxyribonucleic acid
GO: Gene Ontology
GTP: Guanosine triphosphate
ID: Intellectual disability
PPI: Protein-protein interaction
RNA: Ribonucleic acid
SNP: Single nucleotide polymorphism
